# Live-in migrant home care workers in Germany: Stressors and resilience factors

**DOI:** 10.1371/journal.pone.0282744

**Published:** 2023-03-22

**Authors:** Milena Kriegsmann-Rabe, Katja Maus, Nina Hiebel, Constantin Klein, Franziska Geiser

**Affiliations:** 1 Department of Psychosomatic Medicine and Psychotherapy, University Hospital Bonn, Bonn, Germany; 2 Department of Palliative Medicine, University Hospital Bonn, Bonn, Germany; 3 University of Applied Sciences for Social Work, Education and Nursing, Dresden, Germany; National Institute of Public Health: Instituto Nacional de Salud Publica, MEXICO

## Abstract

**Background:**

Thousands of Eastern Europeans find employment caring for older individuals as transmigrating live-in home care workers in private households in Germany. Studies have shown that the stressors threatening their well-being are multifaceted and include inequalities and a high practical and emotional workload, but research on protective factors is still scarce.

**Aim & methods:**

This qualitative descriptive study focuses on both the stressors and factors that promote care workers’ well-being and contribute to their psychological resilience. In guideline-based interviews, 14 female and one male care workers were asked about their stressors and the factors that help them cope.

**Results:**

Identified stressors included separation from their own family, strained relationship with either or both the care recipient (dementia) and their relatives (violation of worker´s rights and devaluation of care work), and permanent availability and lack of free time due to a 24-h care schedule. Resilience factors were both external and internal and included positive social relationships, self-determination, experience in care work, and intrinsic job motivation.

**Conclusion:**

Live-ins reside in an ambiguous setting, exposed to both structural and individual strains. However, external and internal resilience factors contribute to a generally positive attitude toward their job and indicate the agency of this precariously employed group. A socially anchored appreciation of their work and an officially controlled expansion of free time are mandatory to improve the working conditions of live-in care workers.

## Introduction

The phenomenon of care migration to countries of the global north [1, 2] and live-in migrant home care work across welfare states [3] has increased in the last decades. This global rise has been founded among others in by the rapidly aging demographic, the growing number of women in the labor market [4], and structures of social politics and social inequality [5]. The need for domestic elderly care in Germany has also intensified in recent years [6], but to date, health care research on this group is still scarce. To gain first insights into the well-being of live-ins in Germany, this study uses a qualitative descriptive approach (see chapter 3.1). At first, the specific background of live-ins working in Germany will be depicted, to contextualize the field of interest and to make it comparable to other countries and contexts; in a second step, intersectional stressors that potentially affect the groups´ well-being will be outlined, as to date, only less scientific knowledge about it is accessible, as shown in chapter 2. Subsequently and common to qualitative descriptive studies [7] the research interest guiding framework, that is here psychological resilience, is briefly presented.

### Background: Live-in care workers in Germany

Live-ins caring for the elderly in Germany usually transmigrate several months per year between their workplace and their home country. Reliable data on the number of migrant care workers working in private households is lacking, and numbers differ between 150,000 and 300,000 [8] to even 600,000 persons [9], depending on the source. The German government has no figures on this group [10]. Predominantly, women from Poland and other Mid- and East-European countries temporally migrate to German private households to care for the elderly–often without any health care qualification, within unclear legal situations and therefore without any official control [11]. The popular term “24-hour-care workers” used to describe this group hints to their task; as these workers live for several weeks in the household of the person they care for, they are assigned to support the household and assist in multiple ways, such as with personal hygiene, mobility, taking medications, leisure activities, and supervision. Practically and emotionally, they are a complement or even a substitute for the family [12] both during the day and at night (even if, formally, their working time is 8 h a day). It is a “complex activity, consisting of household, care and emotional elements” [13].

### Potential intersectional stressors of migrant home care workers and the aim of this study

Health research on live-ins is scarce. Thus, in the following section potential stressors of related groups will be depicted. Women and gender studies highlight a multitude of inequalities live-ins experience, e.g., a lack of social protection or the emotional and care inequality with regard to the eventuality that caregivers may give less attention to their own relatives in favor of caring for strangers [4]. An intersectional point of view indicates that these care workers, (trans)migrants and most often women, can be affected by diverse stressors. Research refers to the vulnerability of nurses to stress and burnout in professional care settings for the elderly [14]; this may be enhanced in an informal setting without colleagues to talk to and without separation of work and private life. Risk factors for caregiver burden include female sex, residing with the care recipient, social isolation, financial stress, higher number of hours spent caregiving, and lack of choice in being a caregiver [15]. Migration itself can be seen as a critical life incident associated with possible negative consequences for mental health [16]. Green and Ayalon [17] who investigated live-in migrant home care workers in Israel, found severe violations of rights and exposure to work-related abuse (i.e., no vacation and no payment on sick days). Therefore, it is not surprising that health research tends to focus on stressors and strains and remains governed by a “pathogenetic paradigm” [18]. Research, especially in the field of foreign domestic workers, with a special interest in health outcomes is scarce [19]. Emunds and Schacher [20] (2012) concluded that there is a need to understand “which salutogenic factors can be activated” [ibid., p. 67] to promote a good standard of health among this special group of care workers.

This study is guided by a resource-oriented viewpoint that asks for factors that promote care workers’ well-being and contribute to their psychological resilience. Inequalities and strains are neither neglected nor relativized but are instead used as the starting point for the search of possible individual solutions and structural implications. In guideline-based interviews, one male and 15 female care workers were asked which stressors and strains they experienced and which factors contributed to their resilience. This study expands knowledge on the less frequented field of live-in care workers in Germany from a health research perspective by using a qualitative descriptive design. To our knowledge, this is the first study to attempt understanding German-based live-in migrant home care workers´ experienced stressors and resilience factors.

## Resource orientation in health care research on live-ins

To date, the majority of caregiving research refers to negative health aspects and risks, neglecting its positive aspects and the employees’ resources [21]. Only a few studies on migrant live-ins set a spotlight on their potential resources. Iecovich [22] (2011) investigated determinants of job satisfaction among these care workers in Israel. De la Cuesta-Benjumea et al. [23] (2012) looked for escape mechanisms to relieve the burden of care for immigrant caregivers in Spain. Heng et al. [19] (2019) investigated the caregiving experiences, needs, and coping strategies of migrant home care workers in Singapore.

### Psychological resilience

Looking at care workers’ psychological resilience can be another way to enrich the less frequented field of health research on live-ins and to structure the themes inductively and deductively found in data material. This study is part of an interdisciplinary research project on resilience (DFG FOR-2686) that has guided the research interest of the authors. The term resilience is often utilized to describe promising answers to various challenges and crises [24]. Definitions of resilience are diverse [25], and there is still no consensus on how to exactly grasp the phenomenon of resilience [26]. An ongoing discussion in psychology asks whether psychological resilience is a personality trait, an outcome, or a process. That is, one opinion is that a resilient person possesses a certain temporal stable personality trait that helps them to cope with an adverse event [27]. Other approaches understand resilience as a certain outcome of a dynamic process of adaptation to adversity [28]. Recent debate and this study focus on the adaptation process itself and indicates that a multifaceted combination of external and internal factors can influence the way a person copes with an adverse situation [26, 29]. A resilience process thus can be described as the “ongoing result of many processes by which a system’s adaptive capacities, often distributed in networks of interconnected systems, are engaged to restore equilibrium or transform the system so it can continue on with its life and development” [30].

## Materials and methods

### Research methodology, design and ethical approval

To answer the research questions, qualitative guideline-based interviews with live-ins building on two explorative expert interviews, were conducted. This study used a descriptive qualitative design to summarize “the informational contents of data organized in a way that best fits the data” [31]. That is, the description entails the results in everyday language, close to the field of interest and thus provides a comprehensive summary of it. Especially for healthcare environments, it “provides rich descriptive content from the subjects´ perspective” [7]. The outcomes may yield the hypotheses for future phenomenological or grounded theory studies [31], as pointed out in chapter 7. The study was approved by the Ethics Committee of the Medical Faculty of the University of Bonn (No. 028/20).

### Data collection and study inclusion

Two explorative expert interviews with managers of brokerage agencies were conducted in February and March of 2020 (50-min long each) and used to construct an interview guideline for the live-in interviews. These managers arrange the employment contract between East-European care workers and German families and provide administrative support. These interviews provided a profound insight into the living and working conditions of live-ins in Germany and therefore opened the field of interest [32]. From these expert interviews, an initial structure for the interviews with the live-ins emerged. Since the managers’ perspective on important interview questions might differ from the live-ins’ perspective, the interview with the live-ins included an open-ended question to add potentially neglected topics.Furthermore, five of the 15 later interview partners (live-ins) were contacted via the first expert. The other interview partners were addressed through a brokerage agency of a welfare association (n = 5), private contact of Author 1 (n = 3), and snowball system (n = 2). Due to the coronavirus disease 2019 (COVID-19) pandemic, 12 qualitative guideline-based interviews were conducted via telephone. Three interviews were held in person following the acute pandemic safety guidelines. Written declaration of consent was signed by all interviewees. The interviewer ensured through queries that the interviewees understood the objective of the study as well as the procedures of data processing and data protection measures. The interviews with the care workers were held between April and October 2020. The length varied between 20 and 70 min, with a mean of 35 min. Inclusion criteria for the interviews were an age of at least 18 years, working experience as a live-in in Germany for a minimum of one year, and the skill to understand and converse in German. The guideline was pilot-tested with interviewee D1. As no changes were made, this interview was included in the dataset. The audio-taped interviews were transcribed verbatim.

### Data analysis

The transcripts were analyzed qualitatively using focused interview analysis, especially theme-oriented analysis [33]. Coding was performed deductively on the basis of the interview guideline, and inductively, using the MAXQDA software version Analytics Pro 2020 Release 20.4.0 [34]. Author 1 analyzed the interviews and Author 2 validated the coding until agreement on the final code tree was reached. Author 5 and Author 1 discussed the findings for a consensus on the analysis.

### Validity

This research followed the criteria of the Consolidated Criteria for Reporting Qualitative Studies [35]. The interview guideline is provided in this manuscript (see Appendix 1 in S1 File). Referring to Denzin [36] and Patton [37], Carter et al. [38] described the following four types of triangulation: method, investigator, theory, and data source. To improve the validity of this qualitative study, data triangulation was performed by including participants with different sociodemographic backgrounds (see 4.1). Furthermore, there was a triangulation of investigators as researchers from different research backgrounds analyzed the data (Author 1: cultural sciences studies; working field: psychosomatic; Author 2: rehabilitation studies; working field: palliative medicine).

## Results

### Participants

A total of 16 migrant live-in care workers (15 women, one man) took part in this study. One interview was excluded due to a lack of German language skills, which left 14 women and one man. All persons were Polish; the average age of interviewees was about 55 years, ranging from 36 to 68 years. The participants had an average of 12.6 years (range: 4–23 years) of working experience as a live-in in Germany; 12 of the 15 interviewees worked for more than 10 years in Germany (see Table 1 for more information). One interviewee worked without an official contract, one worked for a brokerage agency, four were self-employed, and the majority were family-employed (n = 9)–that is the family acts as the employer. The sample showed a high heterogeneity toward education; the study included people with and without formal professional training (both, n = 6), with a university degree (n = 1), or with unfinished studies (n = 2). Regarding care work experience, the sample was more homogeneous, as just one person had (incomplete) professional training in care work, and the majority had no care work experience prior to working in Germany (n = 6) or just informal care work experience with own family members or neighbors (n = 7). Individual interviewees mentioned a workshop on care work prior to coming to Germany (n = 2).

**Table 1:** Sociodemographic data, work experience and employment status of participants.

*Participants Acronyms*	*Gender*	*Age*	*Home country*	*Education/apprenticeship*	*Experience in care work before Germany*	*Years of experience working as live-in*	*Employment status*
D1	f	54	Poland	No formal professional training	No	11	Self-employed
D2	f	56	Poland	Professional training	No	23	Self-employed
D4	f	57	Poland	Professional training	Family	13	Family-employed
D5	f	37	Poland	University studies	No	10	Self-employed
D6	f	65	Poland	Professional training	Family	12	Self-employed
D7	f	56	Poland	No formal professional training	Older neighbours and experience in retirement home	8	Family-employed
D8	f	51	Poland	Professional training, quit university studies	Family	12	Family-employed
D9	f	56	Poland	Apprenticeship	40 hours workshop on care	10	Family-employed
D10	f	36	Poland	University studies, not finished	No	8	Polish brokerage agency
D11	m	57	Poland	University studies, not finished	Family	4	Family-employed
D12	f	59	Poland	No formal professional training	Family	18	Family-employed
D13	f	68	Poland	No formal professional training	No	12	Family-employed
D14	f	50	Poland	Professional training, not finished	Care work professional training	17	Family-employed
D15	f	65	Poland	Professional training	Family & 30 hours course on dementia/Alzheimer’s	20	Family-employed
D16	f	53	Poland	No formal professional training	No	11	No contract

### Results: Stressors and resilience factors of live-ins

Stressors as well as resilience factors were identified within the interviews. The 15 participants mentioned stressors and strains related to two interdependent spheres: private life and working life. As working life conditions affect the private life of the live-ins and the private situation extends into the working life, separation of the spheres is artificial. Still, to structure this text, they will be depicted separately. Afterwards, the resilience factors and strategies that help live-ins to maintain stability despite these stressors are explored.

#### Private life stressors

Working in another country for several months per year affected the workers’ private lives in their home country. Nine interviewees mentioned stressors with regard to their own family, including separation from their family, children, or partner. As 53-year-old D16 says:

“The worst thing for me was when I spoke to my children, and my child cried. In Germany *I managed every situation*, I *managed everything [italic type = interviewee emphasized words]*, no matter what. Getting up in the nighttime or whatever. But the worst thing is, that my family is not there” (D16, 144ff.).

D5, a 37-year-old mother of two children, feels guilty and explains: “I often go to Germany, also for my children, but I am afraid that my children will say one day: Mom, you were *not* there. And I am afraid of this sentence, and I wish that it will never come [laughs]” (D5, 234ff.).

Furthermore, the intimate relationship with a partner or spouse can be threatened by the separation. After working for 17 years in Germany, 50-year-old D14 stresses that “the separation, when my husband is in Poland and I am not there […] this is slowly getting *too* much for us” (D14, 306ff.).

Four interviewees mentioned the challenge of having their own parents or parents-in-law in need of care at home. For example, D15 needs to organize hour-wise care for her mother in Poland as she is not there to help. This challenging situation takes a lot of her free time, as she always has to coordinate help from far away.

#### Work life stressors

*Stressors in the relationship with the person cared for*: *Cognitive neurodegenerative diseases of the care recipient and multiple challenges*. A wide range of stressors concerning work life was mentioned in the interviews. For instance, 12 of the live-ins reported that their relationship with the person they cared for can contain stressful moments. Primarily, interviewees declared the challenges that come along with diseases of the person they care for, particularly cognitive neurodegenerative diseases, such as Alzheimer’s disease and dementia (n = 9).

For example, 56-year-old D2, who has worked in Germany for 23 years, describes a “terrible” job experience (D2, 120):

“My last patient had […] dementia, Alzheimer’s, and Parkinson […] She was really aggressive and had hallucinations. For example […] she said ´Do you see the dogs on the tree?´[…] And really uncomfortable was when she was completely wet […] she said `I am not dirty, you are dirty´ […] And she did not want me to clean it up. Always a battle […] and she was really aggressive, she wanted to beat me all the time” (D2, 112ff.).

Answering the question, what she would change if she could wish for something regarding her work as live-in, D6, who had 12 years working experience in Germany, said:

“I just wish no Alzheimer’s, that is difficult, and no stroke. I prefer that the person can still stand up a bit, walk a bit by herself, talk a bit, something like that. That is just good” (D6, 312ff.).

D11, the only man in the sample and the person with the least experience in care work in Germany (four years), also answered this question by referring to the challenging aspects of dementia.

Other diseases, such as bulimia, alcoholism, or cancer of the persons cared for, were also mentioned as stressful factors that influence the relationship between the live-in and care recipient and thus affected the live-ins´ well-being.

Participants also spoke about general difficulties with the person they cared for, such as missing sympathy, issues with the older person giving up control, lack of trust of them in the live-in, or a feeling of insecurity and alienation while living in someone else’s household. Singular statements stressed the challenges of being friendly all the time, caring for those of a different gender or age, or those with divergent interests.

#### Stressors in the relationships to the family of the care recipient

Twelve of the 15 interviewees talked about minor or major problems with the family of the person they cared for. These problems consist of violations of the rules and arrangements by the relatives, such as demanding the live-in to cook for the whole family or work in the household of the relatives. Sexual harassment, cultural racism toward Polish workers, and financial fraud were addressed as well. D15, a 65-year-old woman who has worked in Germany for 20 years, talked about the son of a person she has cared for:

“I had a job where I was just half a year. The son of the grandma was really impertinent, and I always had to ask for the money. And he had forgotten it; then he didn’t have it with him [….] At the day of payment, he said I wasn’t there for 24 hours, but just 12 hours. But I said, *I was there*” (D15, 112ff.).

The son ignored the time D15 was at rest or waiting for active tasks; however, this availability time is working time, as another interviewee underlined: “You have to work many hours like a fireman […] The fireman also sits sometimes and plays something. But as soon as there is a call, you have to get up, for example at midnight” (D14, 100ff.). Other relatives of the care patient also lacked a sense of awareness for the complex reality of being a live-in by demanding they stay longer than they wanted to—ignoring their needed family time in Poland.

D10 talked about her first experience as a live-in in Germany. Today, at 36 years old and with a degree, she reflects on her first stay in Germany eight years ago:

“I can still remember very well. I had *no single* day off in six weeks. Because the family did not want to care for their relative, even if they lived right next to the house. They had time, I saw that, but they just did not feel like it” (D10, 138ff.).

Glaring workers’ right violations, like D10 mentioned, as well as simple ignorance of the live-ins needs and wishes were depicted as stressors and strains regarding the relationship to the relatives of care recipients.

#### General stressors of 24-h care work conditions

Being present seven days per week and nearly 24 h per day is an immense stressor for the well-being of care workers. In 12 interviews, live-ins mentioned difficulties caused by a lack of breaks, interrupted night’s rest, a missing private life, the general length of the stay in Germany, or the feeling of isolation.

Women and men working as live-in in Germany usually stay between six and 12 weeks in Germany, followed by a comparable amount of time in their home country, according to the interviews. As an exception, one interviewee stays two months in Germany and just one week to 10 days in Poland (D15). D14 stays four weeks in Germany. Moreover, two of the interviewees permanently stay in Germany, returning to Poland only for holidays (D8 and D9).

D10, for example, explains the feeling of missing privacy and leisure time while being present around the clock:

“The most [difficult] is, that you do not feel so free. That disturbs me the most. I cannot decide on my life, because everything, everything is planned for the person in need of care. That is of course clear. But for example, leisure time, two hours, but you always watch the clock, because you have to do that […] Because you have to be in time for the coffee and you just have to subordinate, yes? You care for other people, that is proper like this, but sometimes it is way too much” (D10, 73–80).

D9, a 56-year-old with 10 years of experience working as a live-in in Germany, emphasized the lack of leisure time as well. Being asked what she would change if she could wish for something, she answers: “My wishes for my work were just a bit more free time [laughs]. Just two hours a day. I have too less time for myself. For example, to sit for myself or doing other things, going to the hairdresser” (D9, 246).

Being the only one responsible for a person in need of care comes with sleeping difficulties. D1 stresses: “During the nights we sleep, yes, but with one ear you hear. Is something going on? Is it not? And that is why you cannot bear it longer [than six weeks]” (D1, 36ff.).

D4, D6, and D14 said that in the beginning of their work in Germany, they miscalculated the time they could bear to stay in Germany: “So when I came for the first time, in 2003, […] my first job was for three months. That were two times three months. And afterwards I said *never again so long*” (D14, 50ff.) and D6 says: “In the beginning I was two months, sometimes three months, but that is too difficult, three months. And the last time I was always six weeks, that is okay” (D6, 15ff.). The interviewees realized how challenging and stressful the time in Germany can be and decided to reduce their stays to an amount that better fits their personal resources.

#### Singularly mentioned burdens

Singularly mentioned burdens for the live-ins were the death of the person cared for, having no friends around, an insufficient payment, own psychological problems, lack of German language skills, feeling of guilt when losing patience in handling persons with dementia, desire for their home country, and no stable place to live, that is not living constantly in either Poland or Germany. Furthermore, several interviewees criticized the brokerage agency that placed them with families. They were reported to not provide sufficient information about the working place, give wrong promises toward the legal status of the interviewee, or exploit women with a lack of German language skills by paying them less than they pay better skilled live-ins. Finally, one interviewee fundamentally criticized the system of brokerage agencies in Germany and Poland in general:

“The Polish agencies work with the German agencies. And this system is close to the border of legality […] There are many juridical problems. […] I hope […] one day our government or the German government will stop the exploitation […] That is a jungle, a real jungle. Everybody does what he can or wants to [laughs]. Of course, that does not get controlled, those are private houses, nobody enters.” (D10, 271ff.).

D10 convincingly addresses the unclear legal situations the live-ins often face that often go along with missing official control [11].

#### Resilience factors of live-ins

Besides the wide range of private and work-related stressors, the interviewees also explained how they coped with challenges and what contributed to their general positive attitude toward the job. These answers can be seen as an expression of the live-ins´ subjective resilience factors. Despite the mentioned challenges, 13 of the 15 interviewees expressed general contentedness with their job. That is, live-ins within different legal conditions—self-employed, family-employed, and brokerage agency-employed—said that they were generally content with their job.

#### Interpersonal resilience factors: Social relationships

An important aspect contributing to this general contentedness and thus to the individuals’ resilience were social relationships.

#### Relatives of care recipient: Appreciation and respect

The relationship with the family of the person cared for and the care recipient is important for the live-ins. As already shown, a strained relationship with these groups can be an enormous stressor; on the other hand, a good relationship can contribute to their contentedness and resilience. Twelve of the interviewed live-ins indicated a generally positive or even very good relationship with the relatives. Good relationships with the relatives were, in these narrations, characterized by being grateful and respectful toward the live-ins and their job. D12 for example is 59 years old and has traveled to Germany for work for 18 years. She intended to work as a live-in as an intermediate solution when she first came to Germany. She had lost her job in Poland and wanted to find something else in her home country. Still, the first family she lived with in Germany played a big role, besides from the financial incentive, in her change of mind:

“For me it was nice, every weekend a son stayed with the mom, and the other one took me in his car, and we jaunted. I was *everywhere* [describes places] […] *Such a nice family*. […] I think, through this family I have decided, to come to Germany again [smirks]” (D12, 183ff.).

Describing her relationship with the family of the person she cares for, D15 states, “The children are friendly. They call, and when we meet they say ´Nice to have you here´” (D15, 206–209).

D6 explains the importance of the families’ respect toward her and her tasks: “The family needs to understand, too, how difficult this work is. […] The family needs to understand, that I need some time, rest time, for myself and they have to understand, this is real work. That is important” (D6, 71ff.).

D6 underlines the importance of a real and true will of the patients´ relatives to understand her, her needs as well as the difficulty of her job.

#### Good functional status of the person cared for and friend- and family-like relationships with the care recipient

Similar to the importance of a good relationship with the relatives of the care recipient, the relationship with the person cared for can not only be a stressor, but also an interpersonal resilience factor. All interviewees mentioned positive aspects of their relationship with the person they care for or have cared for. These aspects include, on the one hand, a good functional status of the care recipient, that is, no Alzheimer’s disease or other forms of dementia, allowing normal communication, and, on the other hand, intimate friend- or family-like relationships, where they could have fun together, learn from each other, talk about problems, and have sympathy for each other in general.

In a detailed description, D16 reports a close relationship with an older woman she had cared for:

“I met a woman who was [high age] […] and I thought, that the work would maybe not be lasting very long, because the lady was already very old. But it was four years. […] And that was a special time. The lady was very wise, very intelligent, and a very cheerful woman, and she is in my heart till today. […] I am really happy that I met these people. I learned a lot and she was really wise and cheerful. […] And I was really happy that I could stay with her till the end” (D16, 89ff.).

D13, a 68-year-old who has worked as a live-in for 12 years, describes a relationship with a care recipient that was family-like: “A single patient I had for *a real long* time. Unfortunately, she died. Six years I had her […] That was nice […] Like a family, a *real family*.” (D13, 151ff.). By repeating the term “family”, D13 expresses the high emotional value of the patient she cared for during a long period.

#### Own family and friends as supportive

Also contributing to the resilience of the live-ins, the relationship with the care worker’s own family and friends was also frequently mentioned (n = 13). Eleven interviewees explained that they call their partners or children in stressful situations. When asked what they commonly do whenever they feel stressed or what they do in concrete stressful situations, the interviewees mentioned the emotional support of their relatives: “Talking to my children and having a good cry” (D15, 176); “Then I call home and call my children” (D13, 163) or “I often spoke to my parents. And they always said ‘Everything will be fine. You’ll come home’ and those words meant a lot to me […] That was a big support” (D10, 156ff.). When asked what gives them strength in their life, seven interviewees explicitly named their family. Singularly, pets were also mentioned as precious, family-like supports.

#### Intrapersonal resilience factors

*Self-determination*. Next to interpersonal factors that are conducive to the live-ins´ resilience, supportive aspects can be found within the individuals themselves. As the foregoing text has shown, a multitude of stressors can affect care workers’ well-being. Still, the abovementioned quotations related to the length of the stay in Germany were influenced by a central element of the live-ins´ possible resilience factors. Despite their often challenging, even stressful working and living conditions, the interviewees showed a high sense of self-determination. Twelve of the 15 participants expressed a strong will, the ability to determine their desired working conditions on their own, and the ability to communicate their wishes and needs.

To repeat D14, for example, dictates the length of her stay in Germany: “So when I came for the first time, in 2003, […] my first job was for three months. That were two times three months. And afterwards I said *never again so long*” (D14, 50ff.). Furthermore, she wants to take control over her leisure time:

“For me, with my experience, I can say that from the beginning in a family, I need more than *other* women. […] When I feel like it, […] I go for a walk in the morning, too. Not just these two hours in the afternoon. When I am looking for a new job, I broach this right from the beginning. I want to *feel* like a *free* human being, too. […] Till now this works” (D14, 106ff.).

Next, to determine the length of the stay in Germany or their leisure time, interviewees also regulate further conditions of their work, such as access to the internet. D11 explained the importance of the internet for corresponding with his family:

“I do always phone to my wife and children. […] I do always have internet and that is important. When I ask the coordinator of the brokerage agency about a new job, I do always ask `Is there internet or not?´. […]The family needs to have internet. I wait […] for one or two weeks, but if there is still no internet, that is impossible” (D11, 187ff.).

In case the working conditions do not fit their expectations, seven interviewees talked about the decision to quit a job. For example, D7 was misinformed by the brokerage agency about the functional status of her next care recipient. She herself is a dainty person but was confronted with the request to care for a man weighing 150 kg.

“I said, ‘I am sorry but I don’t manage to do this.’ […] The coordinator of the brokerage agency replied: ‘You have to, I don’t have anybody else.’ […] I said ‘I don’t have to.’ I take my luggage, wait for the bus and go home […] I just have one back […] and what do *I* do, when it is broken?” (D7, 216ff.).

Responding to relatives of the care recipient that want her to do more than she has agreed to, D7, a 56-year-old who has worked as a live-in for eight years, says: “*I am the care worker here*. […] I am no cleaning lady, no cook, I can do that, but I do not have to” (D7, 356–357) and “I am the care worker for the person in need of care, but *not* for the whole family” (ibid., 374–375), indicating that she strongly sets limits.

In line with this sense of self-determination, D10 stresses:

“You have to take care of your rights. You are a human being too, not a slave. You don´t have to work for the daughter, […] ironing or whatever. When you have a contract, you have to stick to it. And you have to respect other people, but they have to respect you as well” (D10, 149ff.).

D5 accentuates a different dimension of self-determination. She explains that she is responsible for herself and for her decisions:

“The worst thing is indeed the separation from my family. However, you always have to see the glass half-full, not half-empty. Because when I go to Germany, this is my voluntary decision. I go to Germany to earn money and you have to make the time nicer somehow. For example, I can sit here in the corner and cry, because my family is far, far away. I have to make the best of it” (D5, 75ff.).

D8 explains the positive effect her work in Germany has had on her independence: “I am self-reliant. I do not depend on anybody. That gives me strength as well. That got stronger since I left the house” (D8, 780–781).

In a very self-reflective manner, D8 describes the effect of her stays in Germany on herself. Self-reliance, independence and strength are attitudes she thus gains by her work.

#### Care work experience

As aforementioned, the average care work experience of the participants was 12.6 years, and the majority (n = 12) had worked for more than 10 years in Germany. Nine individuals mentioned that their live-in care work experience is supportive in managing their stressful and challenging working and living conditions. Within this time, they became accustomed to their separation from their families, learned how to adapt to new situations, and particularly cultured techniques in handling persons with dementia, Alzheimer’s disease, or other diseases. D11, for example, cared for a person with cancer, which required him to perform mucus aspiration occasionally—a work he had never done before: “That was really difficult. I had to watch out […] There was a lot of mucus. I always had to do a mucus aspiration. […] But now I have big experience [smirks]” (D11, 121ff.).

D10 explains the habituation that comes along with years of experience: “In the beginning, I had no idea how it [the job as a live-in] works. But as I said, the work is similar, and with time practice comes and you are used to it” (D10, 89–91).

D7 describes what she has learned while caring for people with dementia:

“The main thing is, you have to be patient. […] You always have to try to use medium tones or a bit calmer. And learn. Learn and every day there is something new. For people with dementia, Alzheimer’s or something else like Parkinson, the people go to bed, but you don’t know about tomorrow. What is the problem the next day?” (D7, 85ff.).

In addition, D15, who reported about the care recipients’ relative who did not pay her on time and ignored availability time as working time, resumes: “I just thought at that time, I will pick my next job in a better way. And if there is a relative […] who seems strange to me in the first talk, I will not do it” (D15, 146–148).

#### Constructively using leisure time

Using the restricted leisure time in a way that is best for them can be seen as a learned experience or adaptation technique that helps live-ins to cope with their stressful everyday life. When asked what they do in their restricted leisure time, interviewees enumerated a long list of hobbies. Next to meeting friends, reading, and listening to music, especially physical movement, such as going for a walk or riding a bike, was mentioned most often (n = 13). D16 illustrates: “I am really active. I do jogging, and the most important for me is having a bike [smirks] […] Movement […], going for a walk, riding a bike, jogging, yes, that’ s the best for me” (D16, 115ff.).

When asked what she does whenever she feels stressed, D14 replied: “I go for a walk […] I need the air” (D14, 199ff.). “I have to go for a walk every day, always. I do Nordic Walking, I ride a bike” (ibid., 92–93). And D12 explains: “I like walking, going for a walk, that’s good for me. Refueling oxygen [smirks]” (D12, 120–121).

Leaving the house, to move around, not looking for someone but for himself or herself and to get some freedom can be considered a resilience strategy. At the same time, it mirrors a main stressor: being present in the house of the person cared for 24 h (except for a 2-h break). Going for a walk every day reflects the limited resources the live-ins do have, as well as the experience and agency to care for themselves.

#### Intrinsic job motivation

Participants’ motivation to work in Germany as a live-in was most often financial stimulus (n = 14), demonstrated by occupational hopelessness in Poland, the wish to renovate or buy an apartment or house, debts, an insufficient pension, and, very often (n = 8), the need to financially support children and grandchildren. Next to this extrinsic motivation, eight of these interviewees additionally mentioned an intrinsic motivation: enjoyment of their work, altruism, and the meaningfulness of their job. Intrinsic motivation can be seen, according to these interviews, as a resource in handling adverse working conditions. Six interviewees explained that they like helping others.

D11: “I *like* helping these people, these sick people. […] I feel very good, when I help and these people are content” (D11, 42–44).

D4 explains the combination of extrinsic and intrinsic motivation:

“This job means a lot of stress. Because money is not everything. I do this job not just because of the money, it’s just that this job makes me happy. Because I can do something for somebody, helping a bit” (D4, 208ff.).

Similar to D4, D2 says:

“If I didn’t have fun or feel like it at work, I would never do this job. But I have a heart like a barn, as my mother said […] [smirks]. That is why I work with sick people, because I think, that is the right thing. It is better than cleaning, because cleaning, that are dead things. People are people. There I know that I did something good for them. And that’s a giant satisfaction for me” (D2, 212ff.).

Furthermore, as eight interviewees mentioned, the extrinsic motivation to financially support their children and grandchildren can also be read as an expression of altruism directed to their family, as can be seen in the following quotation from D5, who answers what gives her strength in her life:

“I think, my family, because I have a goal. I came to Germany to reach my target. That is money in this case. And I think when I got the money, I can offer something better to my children, like a house or […] a new laptop. […] All that what I didn´t have, I can offer to my family” (D5, 142ff.).

D5 wishes to fulfil material wishes to her family. The job as live-in in Germany offers her the option to do so.

#### Singularly mentioned resilience factors

Less often mentioned were the following intra- and interpersonal factors that positively contribute to the live-ins´ resilience: their own health sensitivity (e.g., “I still don´t have any health problems, that might be important as well,” D12, 204–205), sufficient German language skills as the premise to express self-determined wishes and needs, external professionals supporting live-ins by doing night-shifts or cleaning, and religiosity/spirituality.

## Discussion

Live-in migrant home care workers in Germany live and work in an ambiguous setting. On the one hand, they are confronted with a wide range of individual and structural stressors that might affect their well-being. On the other hand, intrapersonal and interpersonal resilience factors can mediate a subjective positive attitude toward their job.

The participants of this study differed from the majority of live-ins in Germany in that nine individuals of the sample were officially employed by the family—contrary to the majority who are not permanently employed [5]. That is, most participants in this study have an official contract with the family, who acts as the employer by registering the live-in and paying their social security contributions [39]. Furthermore, five of the six other interviewees also have a legal status as self-deployed, or brokerage agency employed. Still, legal employment relationships do not protect live-ins from immense family life- and work-related stressors:

The spatial separation from children, partners, and parents is the main stressor with regard to the private life of the live-ins. Participants miss their family and feel guilty toward children left behind and responsible for older relatives in need of care. This finding supports the criticism toward structurally caused “emotional and care inequality” [4]. This is an implicit facet of migrant home care work, as the provision of physical care is mainly restricted to the care recipient in Germany and less intense to the family back home [ibid.].

In line with the findings of Heng et al. [19] who investigated foreign domestic workers caring for older people in Singapore, dementia-related behavioral issues of care recipients were found as a major stressor regarding working conditions. Even though the participants had worked an average of more than 10 years as a live-in, dementia, such as Alzheimer’s disease, was often mentioned as an immense stressor. Apparently, a complete habituation toward challenges caused by these diseases is not possible over time, and cognitive-emotional impairment of the person cared for has to be seen as a permanent stressor. These frequently found findings point towards the fact that live-ins in Germany do not get any adequate training in the care of people with cognitive neurodegenerative diseases and do not feel profoundly prepared for these tasks. Similar to the findings of Kiekert and Schirilla [40] these outcomes highlight the need for a transfer of knowledge to be fostered on the organizational level. Information on the functional status of the care recipients needs to be provided by the agencies and/or families prior to the job start; a theoretical and practical training as well as the permanent offer to get support in emergency situations need to be institutionalized.

The relationship with the family of the care recipient can be challenging as well. In line with other studies, violations of rules and workers’ rights [17, 41] were found, as well as a lack of respect toward the care workers and a lack of appreciation for their work [41, 42]. That is, relatives of the care recipient did not acknowledge the availability time the live-ins provide, e.g., during the night, or the needs of the live-ins such as sufficient time with their own family.

In general, being present nearly 24-h a day is a major stressor affecting the live-ins´ well-being. This permanent availability comes with limited nighttime rest, a lack of free time, and restrictions on their private life. This is consistent with challenges reported by other migrant home care workers [19, 40] and can co-cause care worker burden [15].

These daily hassles and structural difficulties challenge the live-ins´ resilience. This study supports the recent direction concerning the definition of resilience, that a multitude of internal and external factors can contribute to a resilient process [26, 29].

In line with other researchers, these findings highlight the importance of positive social relationships to the well-being of live-in care workers. A good relationship with the relatives of the care recipients based on respect and appreciation of the caring person and their work is fundamental to their well-being [19, 40, 41]. Live-in care workers and care recipients may even develop a family- or friend-like relationship [43, 44]. An important requirement for this type of relationship is the functional status of the care recipient, that is, preferably no degenerative brain diseases. Finally, the emotional support of the live-ins´ family in the home country is mentioned to be a major external resilience factor [19].

Predominant within the interviews was a sense of self-determination of the participants. They set limits regarding their working conditions, quit if they did not fit, communicated their wishes, and understood themselves to be independent human beings. Work experience also supported the live-ins´ well-being, as adaptation techniques and strategies toward challenges can be cultivated. The target-oriented utilization of restricted leisure time [44] is considered as a learned experience that helps live-ins to cope with work-related stressors. Furthermore, beside the financial motivation, participants also showed an intrinsic motivation toward this type of work, which consists of altruism toward the care recipients as well as their own family. The live-ins often like to care for other persons and appreciate the role of breadwinner for their family back home. Thus, regarding the internal resilience factors, this study is in line with other researchers who highlight the agency or autonomy of live-ins and individual care work gains within stressful working conditions [5, 13, 45, 46]. In accordance with the self-determination theory [47], the intrinsic motivation characterized by a sense of choice and personal values helps the live-ins maintain mental health and personal well-being.

Psychological resilience, according to these findings, is based upon both external (social and environmental) and internal (traits, experiences, and motivations) factors. Reducing the phenomena of resilience to singular traits or outcomes does not, in our opinion, adequately reflect its complexity.

## Limitations and strengths

To our best knowledge, this research is the first to access both stressors and resilience factors of German-based live-in migrant home care workers. Still, as is usual in qualitative research, the findings of this study do not claim representability and therefore cannot be generalized. The participants of the study had sufficient German skills and mostly lived within legal conditions—different from other individuals who come to Germany without any language skills or contract. Only one interviewee did not have any official status, that is, she was not registered as a care worker by either the family, a brokerage agency, or herself. As the COVID-19-pandemic appeared right after the start of the study, interviews had to be conducted via telephone (except for three), which was different from the atmosphere of conversations held in person. Still, this factor might have positively contributed to the willingness to participate, as interviews could take less of the interviewees’ limited free time. All interviewees came from Poland, so statements about persons from other countries cannot generally be concluded from the findings. Five of the 15 interviewees were contacted via one brokerage agency, and five were affiliated with a welfare association. The former was responsible for the high percentage of participants who were family-employed as this brokerage agency exclusively works with family-employer contracts. Statements about the impact of the brokerage agencies on the answers of participants cannot be made; still, to achieve as open a conversation as possible, the interviewer, Author 1, underlined the anonymity of the interview before the beginning. In addition, the majority of the live-ins has a long care work experience (more than 10 years)–statements on the representability of the findings for women and men with less experience cannot be made. We were aware that a conflict of interest could be assumed in the expert interviews with the managers who, on the one hand, are interested in better conditions for live-ins, but on the other hand also have financial interests in this employment model. However, these managers have the largest overview of the living and working conditions of live-ins in Germany, and the expert interviews were helpful for an exploration of the field and the creation of the interval guide. An open question was included in the guide to allow the live-ins interviewed to fill in previously missing topics.

## Conclusion and practical implications

Home care workers do not have the same level of protections by workplace safety policies such as those in hospitals and nursing homes [48]; this is especially true for live-in migrant home care workers. Our findings emphasize the importance of support groups and/or profound trainings that provide live-ins with information and practical techniques with regard to degenerative brain diseases or other illnesses and specific care needs [49]. As this study has shown, positive social relationships are essential for live-in care workers’ well-being, for which both respect and appreciation of live-ins and their work are crucial. Thus, brokerage agencies, researchers, and the public media should raise awareness among families of care recipients to value this work. Mandatory for all implications is a substantive expansion of leisure time and privacy for care workers—as the given structural inequalities of 24 h of care work seven days per week are not, per se, compatible with workers’ rights. Central to this transformation is a change in social politics of the German government and society, which relies on migrant care workers but does not adequately care for them. A characteristic of this study´s design, a qualitative descriptive approach, is that it stays closer to the data than grounded theory or phenomenological studies [31]–still, the study´s outcomes can be used as starting point for future qualitative studies focusing e.g. care worker/care recipient relationships or implications for health policy and practice.

## Supporting information

S1 File Appendix 1: Interview guideline Live-ins. (DOCX)

S2 File COREQ. (DOCX)
