# Supplementary material for: Live-in migrant home care workers in Germany: Stressors and resilience factors
Source: PLoS One. 2023 Mar 22;18(3):e0282744. doi: 10.1371/journal.pone.0282744 (PMC10032493; doi:10.1371/journal.pone.0282744)
Supplement: S1 File — (DOCX) [file pone.0282744.s001.docx]

**Appendix 1: Interview guideline Live-ins**

1. How long have you been working as a caregiver in Germany?

- Did you have any experience in care work before you came here?
- How long do you regularly stay in Germany?
- What motivates you to work in Germany?

2. What does a typical working day look like?

- Which difficulties do you face in your daily work?
- How do you cope with these difficulties?

3. How do you spend your free time?

4. Would you recommend the work as caregiver in Germany to a friend/your children?

- Why, why not?
- What would be tips you would give him/her?

5. When you feel stressed or sad in Germany, what is the first thing you do?

- Is there someone you can talk to about your problems?

6. What was the worst experience during your time in Germany and how did you deal with it?

7. What gives you strength in your life?

8. What are you most looking forward to at home?

9. What are you not so much looking forward to at home?

10. Will you continue to work as a caregiver in Germany in the future?

11. If you could wish for one thing to be different about your work, what would that be?

12. How would you like to be cared for when you are old?

13. Is there anything else you would like to add?
