# Supplementary material for: Live-in migrant home care workers in Germany: Stressors and resilience factors
Source: PLoS One. 2023 Mar 22;18(3):e0282744. doi: 10.1371/journal.pone.0282744 (PMC10032493; doi:10.1371/journal.pone.0282744)
Supplement: S2 File — (DOCX) [file pone.0282744.s002.docx]

**COREQ (TONG ET AL. 2007)**

(32-item checklist, consolidated criteria for reporting qualitative studies)

**INHALT**

[1. Research teAm and reflexivity 2](#_Toc44676421)

[1.1 Personal Characteristics 2](#_Toc44676422)

[1.1.1 Interviewer/facilitator 2](#_Toc44676423)

[1.1.2 Credentials 2](#_Toc44676424)

[1.1.3 Occupation 2](#_Toc44676425)

[1.1.4 Gender 2](#_Toc44676426)

[1.1.5 Experience and training 2](#_Toc44676427)

[1.2 Relationship with participants 2](#_Toc44676428)

[1.2.1 Relationship established 2](#_Toc44676429)

[1.2.2 Participant knowledge of the interviewer 2](#_Toc44676430)

[1.2.3 Interviewer characteristics 2](#_Toc44676431)

[2. Study design 3](#_Toc44676432)

[2.1 Theoretical Framework 3](#_Toc44676433)

[2.1.1 Methodological orientation and Theory 3](#_Toc44676434)

[2.2 Participant selection 3](#_Toc44676435)

[2.2.1 Sampling 3](#_Toc44676436)

[2.2.2 Method of approach 3](#_Toc44676437)

[2.2.3 Sample size 3](#_Toc44676438)

[2.2.4 Non-participation Setting 3](#_Toc44676439)

[2.3 Setting 3](#_Toc44676440)

[2.3.1 Setting of data collection 3](#_Toc44676441)

[2.3.2 Presence of non-participants 4](#_Toc44676442)

[2.3.3 Description of sample 4](#_Toc44676443)

[2.4 Data collection 4](#_Toc44676444)

[2.4.1 Interview guide 4](#_Toc44676445)

[2.4.2 Repeat interviews 4](#_Toc44676446)

[2.4.3 Audio/visual recording? 4](#_Toc44676447)

[2.4.4 Field notes 4](#_Toc44676448)

[2.4.5 Duration 4](#_Toc44676449)

[2.4.6 Data saturation 4](#_Toc44676450)

[2.4.7 Transcripts returned 4](#_Toc44676451)

[3. Analysis and findings 4](#_Toc44676452)

[3.1 Data analysis 4](#_Toc44676453)

[3.1.1 Number of data coders 4](#_Toc44676454)

[3.1.2 Description of the coding tree 4](#_Toc44676455)

[3.1.3 Derivation of themes 4](#_Toc44676456)

[3.1.4 Software 4](#_Toc44676457)

[3.1.5 Participant checking 4](#_Toc44676458)

[3.2 Reporting 4](#_Toc44676459)

[3.2.1 Quotations presented 4](#_Toc44676460)

[3.2.2 Data findings consistent 5](#_Toc44676461)

[3.2.3 Clarity of major themes 5](#_Toc44676462)

[3.2.4 Clarity of minor themes 5](#_Toc44676463)

# Research team and reflexivity

## 1.1 Personal Characteristics

### 1.1.1 Interviewer/facilitator

*Which author/s conducted the interview?*

Author 1 was responsible for this exercise. Author 5 took part in the process of creating the interview guidelines.

### 1.1.2 Credentials

*What where the researcher`s credentials? E.g. PhD, MD*

Author 1 is Cultural Anthropologist, M.A. and therefore trained in conducting qualitative interviews. Author 2 holds a master in Rehabilitation Studies, Author 3 is psychologist, PhD; the discipline of Author 4, professor, are theology and psychology; those of Author 5, professor as well, are medicine and psychology.

### 1.1.3 Occupation

*What was their occupation at the time of the study?*

Author 1 was and is PhD-student at the Medical faculty of the University of Bonn and research assistant at the Department of Psychosomatic Medicine and Psychotherapy, University Hospital Bonn. Author 2 was and is PhD-student at the Medical faculty of the University of Bonn and research assistant at the Department of Palliative Medicine, University Hospital Bonn. Author 3 was post-doc at the Department of Psychosomatic Medicine and Psychotherapy, University Hospital Bonn. Author 4 was and is professor at the University of Applied Sciences for Social Work, Education and Nursing, Dresden. Author 5 was and is clinical director of and professor at the Department of Psychosomatic Medicine and Psychotherapy, University Hospital Bonn. All authors are part of the same interdisciplinary research group on individual resilience.

### 1.1.4 Gender

*Was the researcher male or female [or divers; M.R.]?*

Except for Author 4 who is male, all researchers are female.

### 1.1.5 Experience and training

*What experience or training did the researcher have?*

Conducting qualitative interviews was an integral part of Author 1`s studies as well as her occupation at the University Hospital of Bonn.

## 1.2 Relationship with participants

### 1.2.1 Relationship established

*Was a relationship established prior to study commencement?*

No relationship with the participants was established prior to study commencement.

### 1.2.2 Participant knowledge of the interviewer

*What did the participants know about the researcher? E.g. personal goals, reasons for doing the research?*

Prior to the interviews, Author 1 explained herself and her personal goals for doing this research. This is most importantly her own experience with East-European live-in caregivers ten years ago, when several women cared for her grandmother. At that time, Author 1 was wondering how these women were capable of doing this challenging job far away from home. As Author 1 works in a resilience research group at the time of the interviews, she also explained her professional motivation to find out more about subjective resilience factors.

### 1.2.3 Interviewer characteristics

*What characteristics were reported about the interviewer/facilitator? E.g. Bias, assumptions, reasons and interests in the research topic*

The interviewer explained her personal experiences with the field of interest (see 1.2.2), the topic of her PhD-project as well as resilience as the object of the research group she was participating.

# Study design

## 2.1 Theoretical Framework

### 2.1.1 Methodological orientation and Theory

*What methodological orientation was stated to underpin the study? E.g. grounded theory, discourse analysis, ethnography, phenomenology, content analysis*

This study used a descriptive qualitative design to summarize “the informational contents of data organized in a way that best fits the data” (Sandelowski 2000, 339). That is, the description entails the results in everyday language, close to the field of interest and thus provides a comprehensive summary of it. The transcripts were analyzed qualitatively using focused interview analysis, especially theme-oriented analysis (Kuckartz & Rädiker, 2020). Coding was performed deductively on the basis of the interview guideline, and inductively, using the MAXQDA software version Analytics Pro 2020 Release 20.4.0 (Verbi Software, 2020).

## 2.2 Participant selection

### 2.2.1 Sampling

*How where participants selected? E.g. purposive, convenience, consecutive, snowball*

The two experts who were interviewed first and the live-ins were selected on purpose. These first interviews provided a profound insight into the living and working conditions of live-ins in Germany and therefore opened the field of interest (Bogner et al., 2014). Furthermore, five of the 15 later interview partners (live-ins) were contacted via the first expert. The other interview partners were addressed through a brokerage agency of a welfare association (n = 5), private contact of Author 1 (n = 3), and snowball system (n = 2).

### 2.2.2 Method of approach

*How where participants approached? E.g. face-to-face, telephone, mail, email*

The two expert interviews were held face-to-face. Due to the coronavirus disease 2019 (COVID-19) pandemic, 12 qualitative guideline-based interviews were conducted via telephone. Three interviews were held in person following the acute pandemic safety guidelines.

### 2.2.3 Sample size

*How many participants were in the study?*

Two expert interviews as access to the field and preparation of the guideline, 16 participants, one interview was kept out of the analysis because the language quality was considered too low.

### 2.2.4 Non-participation Setting

*How many people refused to participate or dropped out? Reasons?*

Two persons refused to participate as they did not have enough time or did not feel comfortable with an interview in German language.

## 2.3 Setting

### 2.3.1 Setting of data collection

*Where was the data collected? E.g. home, clinic, workplace*

The expert interviews were held at the workplace, the office of the participants. Four live-ins were at home during the telephone interviews, 12 were at their workplace.

### 2.3.2 Presence of non-participants

*Was anyone else present besides the participants and researchers?*

Nobody else was present besides the participants and the researcher.

### 2.3.3 Description of sample

*What are the important characteristics of the sample? E.g. demographic data, date*

**Tab. 1 Sociodemographic data, work experience and employment status of participants**

| *Participants Acronyms* | *Gender* | *Age* | *Home country* | *Education/apprenticeship* | *Experience in care work before Germany* | *Years of experience working as live-in* | *Employment status* |  |
| --- | --- | --- | --- | --- | --- | --- | --- | --- |
| D1 | f | 54 | Poland | No formal professional training | No | 11 | Self-employed |  |
| D2 | f | 56 | Poland | Professional training | No | 23 | Self-employed |  |
| D4 | f | 57 | Poland | Professional training | Family | 13 | Family-employed |  |
| D5 | f | 37 | Poland | University studies | No | 10 | Self-employed |  |
| D6 | f | 65 | Poland | Professional training | Family | 12 | Self-employed |  |
| D7 | f | 56 | Poland | No formal professional training | Older neighbours and experience in retirement home | 8 | Family-employed |  |
| D8 | f | 51 | Poland | Professional training, quit university studies | Family | 12 | Family-employed |  |
| D9 | f | 56 | Poland | Apprenticeship | 40 hours workshop on care | 10 | Family-employed |  |
| D10 | f | 36 | Poland | University studies, not finished | No | 8 | Polish brokerage agency |  |
| D11 | m | 57 | Poland | University studies, not finished | Family | 4 | Family-employed |  |
| D12 | f | 59 | Poland | No formal professional training | Family | 18 | Family-employed |  |
| D13 | f | 68 | Poland | No formal professional training | No | 12 | Family-employed |  |
| D14 | f | 50 | Poland | Professional training, not finished | Care work professional training | 17 | Family-employed |  |
| D15 | f | 65 | Poland | Professional training | Family & 30 hours course on dementia/Alzheimer’s | 20 | Family-employed |  |
| D16 | f | 53 | Poland | No formal professional training | No | 11 | No contract |  |

## 2.4 Data collection

### 2.4.1 Interview guide

*Were questions, prompts, guides provided by the authors? Was it pilot tested?*

The guideline for the live-in interviews was pilot-tested with interviewee D1. As no changes were made, this interview was included in the dataset.

Interview guideline Live-ins

1. How long have you been working as a caregiver in Germany?

- Did you have any experience in care work before you came here?
- How long do you regularly stay in Germany?
- What motivates you to work in Germany?

1. What does a typical working day look like?

- Which difficulties do you face in your daily work?
- How do you cope with these difficulties?

1. How do you spend your free time?
2. Would you recommend the work as caregiver in Germany to a friend/your children?

- Why, why not?
- What would be tips you would give him/her?

1. When you feel stressed or sad in Germany, what is the first thing you do?

- Is there someone you can talk to about your problems?

1. What was the worst experience during your time in Germany and how did you deal with it?
2. What gives you strength in your life?
3. What are you most looking forward to at home?
4. What are you not so much looking forward to at home?
5. Will you continue to work as a caregiver in Germany in the future?
6. If you could wish for one thing to be different about your work, what would that be?
7. How would you like to be cared for when you are old?
8. Is there anything else you would like to add?

### 2.4.2 Repeat interviews

*Were repeated interviews carried out? If yes, how many?*

There were no repeated interviews.

### 2.4.3 Audio/visual recording?

*Did the research use audio or visual recording to collect the data?*

Audio recording was used to collect the data.

### 2.4.4 Field notes

*Were field notes made during and/or after the interview?*

Both during the interview, small bullet points, including follow-up questions were made and an interview protocol with memo on atmosphere and special features was made after each interview.

### 2.4.5 Duration

*What was the duration of the interviews?*

The expert interviews were each 50 minutes long. The length of the live-in interviews varied between 20 and 70 min, with a mean of 35 min.

### 2.4.6 Data saturation

*Was data saturation discussed?*

When no essential new themes derived from the interviews, the recruiting was stopped.

### 2.4.7 Transcripts returned

*Were transcripts returned to participants for comment and/or correction?*

Transcripts were not returned to participants for comments/correction.

# Analysis and findings

## 3.1 Data analysis

### 3.1.1 Number of data coders

*How many data coders coded the data?*

Author 1 analyzed the interviews and Author 2 validated the coding until agreement on the final code tree was reached. Author 5 and Author 1 discussed the findings for a consensus on the analysis.

### 3.1.2 Description of the coding tree

*Did authors provide a description of the coding tree?*

If readers are interested, the coding tree can be translated from German to English.

### 3.1.3 Derivation of themes

*Were themes identified in advance or derived from the data?*

Coding was performed deductively on the basis of the interview guideline, and inductively.

### 3.1.4 Software

*What software, if applicable, was used to manage the data?*

MAXQDA software version Analytics Pro 2020 Release 20.4.0 (Verbi Software, 2020) was used.

### 3.1.5 Participant checking

*Did participants provide feedback on the findings?*

Due to the restricted time of the PhD-project of Author 1, we unfortunately could not offer participants to provide feedback on the findings. However, all participants will get access to the results after they are published.

## 3.2 Reporting

### 3.2.1 Quotations presented

*Were participant quotations presented to illustrate the themes/findings? Was each quotation identified? E.g. participant number*

Participant quotations are presented frequently to illustrate the themes. Each quotation is identified by a participant number.

### 3.2.2 Data findings consistent

*Was there consistency between the data presented and the findings?*

We strived to show the consistency between the data presented and the findings. Readers have to decide upon the quality.

### 3.2.3 Clarity of major themes

*Were major themes clearly presented in the findings?*

Each major theme was clearly presented in the findings by a separate paragraph and title.

### 3.2.4 Clarity of minor themes

*Is there a description of diverse cases or discussion of minor themes?*

We focussed on major themes in our article; however, for disclosure we provide a paragraph, titled “Singularly mentioned burdens” and “Singularly mentioned resilience factors”.
